# The complications associated with Resuscitative Endovascular Balloon Occlusion of the Aorta (REBOA)

**DOI:** 10.1186/s13017-018-0181-6

**Published:** 2018-05-11

**Authors:** Marcelo A. F. Ribeiro Junior, Celia Y. D. Feng, Alexander T. M. Nguyen, Vinicius C. Rodrigues, Giovana E. K. Bechara, Raíssa Reis de-Moura, Megan Brenner

**Affiliations:** 1 Disciplina de Cirurgia Geral e Trauma, Universidade Santo Amaro, São Paulo, São Paulo Brazil; 2 School of Medicine, University of New South Wales, Sydney, New South Wales Australia; 3 RA Cowley Shock Trauma Center, University of Maryland, Baltimore, MD USA

**Keywords:** Complications, Radiology, Interventional, Multiple trauma, Abdomen, Shock, Hemorrhagic, REBOA

## Abstract

Non-compressible torso hemorrhage (NCTH) remains a significant cause of morbidity and mortality in the field of trauma and emergency medicine. In recent times, there has been a resurgence in the adoption of Resuscitative Endovascular Balloon Occlusion of the Aorta (REBOA) for patients who present with NCTH. Like all medical procedures, there are benefits and risks associated with the REBOA technique. However, in the case of REBOA, these complications are not unanimously agreed upon with varying viewpoints and studies. This article aims to review the current knowledge surrounding the complications of the REBOA technique at each step of its application.

## Background

**Fig. 1:**
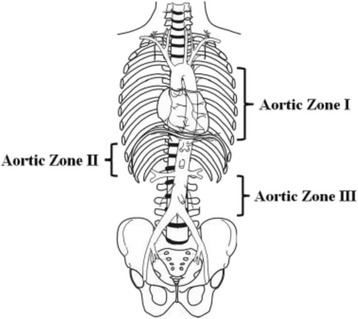
The aortic zones in relation to the REBOA procedure. Retrieved from Stannard, Eliason [8]

Non-compressible torso hemorrhage (NCTH) is a major cause of morbidity and mortality in the trauma setting [1]. The difficulty in controlling NCTH arises from the fact that the bleeding cannot be managed like other types of traumatic hemorrhage, such as the use of tourniquets or direct pressure in limb hemorrhage [2, 3]. Instead, highly invasive techniques such as resuscitative thoracotomies (RT) are used to control thoracic bleeding. RT has low rates of patient survival as well as increased exposure of health care workers to blood-borne pathogens [4, 5]. Resuscitative Endovascular Balloon Occlusion of the Aorta (REBOA) is an old technique that has been receiving renewed interest in recent years [1, 6]. As the name suggests, the technique involves the introduction of a balloon occlusion catheter via the femoral artery into the aorta and inflating the balloon at one of two aortic zones (zone I or zone III) depending on the circumstances [7, 8]. The aorta can be divided into three zones (Fig. 1): with zone I being the aorta between the let subclavian artery and the celiac trunk, zone II being the aorta between the celiac trunk and the lowest renal artery, and zone III being the area between the lowest renal artery and the aortic bifurcation [8]. Zone II is not for occlusion [8]. The balloon is then inflated to stem the flow of blood and later deflated and removed [8]. Renewed interest particularly in the USA in REBOA has led to its introduction in many trauma centers, as well as increased levels of research and analysis regarding the technique [9].

REBOA shows promise in improving the outcomes for patients with NCTH in comparison to RT. In a recent prospective study, there was no significant difference in overall mortality between patients undergoing RT and those undergoing REBOA for NCTH (REBOA, 71.7 vs. RT, 83.8%; *p* = 0.120) [9]. However, there are also complications associated with the procedure. Excessive ischemia during aortic occlusion, post-operative thrombosis, and limb amputation are among some of the reported complications of the procedure [10].

## Methods

Considering the increasing number of cases treated using the REBOA technique, the aim of this paper is to review complications of REBOA at each stage of the procedure using a combination of literature review and clinical experience. PubMed online searches were used including search words such as REBOA, resuscitation, hemorrhage, and shock. We also highlight key areas for further investigation and research.

## Results

### Arterial access and balloon positioning

Complications of REBOA are numerous and can be caused by the insertion of the intra-aortic balloon occlusion catheter and femoral artery sheath. The major complications of REBOA catheter insertion are vessel injuries (aortic dissection, rupture, and perforation), embolization, air emboli, and peripheral ischemia [11].

The greatest limitation to REBOA is the ischemia caused by total aortic occlusion [12]. Prolonged ischemia followed by reperfusion can result in multiple organ failure including acute kidney injury, liver failure, spinal cord infarction, intestinal ischemia, myonecrosis, limb loss, and death [12, 13].

The severe ischemic complication of the lower extremities can be associated with sheath placement for REBOA, and the use of large-sized sheaths for REBOA can be a critical risk factor for lower extremity ischemia [13].

As blood flow is inversely proportional to the vessel cross-sectional area, it is acceptable that large-sized sheaths may decrease blood flow to the extremities [13]. Some experts recommend first accessing the artery with a 4–5 Fr micropuncture catheter, suggesting that the smaller sheath can be used proactively in patients who may deteriorate, allowing for arterial blood pressure monitoring and collection of blood samples. Then, the micropuncture catheter should be rapidly exchanged for a 7–8 Fr sheath via the Seldinger technique for REBOA access with a relatively low risk of serious complications [14].

REBOA has been used routinely in endovascular management of abdominal aortic aneurysms (EVAR) via large diameter sheaths, typically 12–14 Fr or larger. The development of balloon catheters deliverable via 7 Fr sheaths have led to new enthusiasm for the technique for trauma patients. However, the evidence for its efficacy is limited [12]. Smaller sheaths appear to have fewer complications despite relatively prolonged placement and require external compression on removal [15].

Although these complications are related to sheath insertion and are not specific to REBOA, it is important for surgeons performing REBOA to be aware of these potential access site consequences and address them at time of sheath removal to avoid limb-threatening vascular complications [16]. Moreover, REBOA should be performed by an acute care surgeon or an interventionalist (vascular surgeon or interventional radiologist) trained in REBOA and, in order to resolve possible vascular complications, a vascular surgeon must be available [17]. When performed by an emergency medicine physician, an acute care surgeon or interventionalist should be immediately available to perform definitive hemorrhage control.

An additional challenge of REBOA is the need for rapid and accurate placement. This technique can provide total occlusion of the aorta either just above the diaphragm (zone I), to control intra-abdominal bleeding, or above the aorto-iliac bifurcation (zone III), to control bleeding in the pelvis or proximal extremities [12, 18].

Animal studies suggest that zone I REBOA is survivable for 60 min and zone III for 90 min. However, the Norii registry study shows that zone I occlusion for 45 min was uniformly lethal and there were only two survivors after 90 min of REBOA occlusion in the Inoue registry study. Once the REBOA catheter is inflated, the time to obtain definitive control of bleeding is limited and the need is absolute [12].

### Balloon inflation

Balloon inflation is an integral part of the procedure and must be executed carefully. The balloon should be inflated until the blood pressure is augmented and contralateral femoral pulse is stopped, approximately 8 mL for zone I or 3 mL for zone III [19].

It is crucial that the practitioner performing REBOA is aware of the complications related to the inflation level and duration of the inflation in this step of the procedure. The physician should be careful to not over-inflate the balloon, as an over-inflation will rupture the balloon or the blood vessel [19]. A systematic review conducted by Morrison JJ et al. in 2016 identified a total of 83 studies which reported three deaths directly associated with balloon-related complications [20]. All patients were being treated for ruptured abdominal aortic aneurysm (rAAA) and had transbrachial aortic occlusion performed. Two balloons ruptured, resulting in precipitous cardiovascular collapse and death. The aortic injury occurred in the setting of postpartum hemorrhage (PPH) and was promptly recognized because of hypotension after hysterectomy and balloon deflation. It was suspected that balloon over-inflation had caused injury to the aorta [20]. As previously mentioned, in order to avoid balloon rupture, the physician must be attentive to the blood pressure and contralateral femoral pulse checking if the first one augmented and the second one stopped [19].

Another complication that must be avoided is the profound ischemia related to a long-term occlusion. Animal data suggests that prolonged occlusion of the aorta is associated with ischemia-reperfusion injury and potentially an increased risk of death [21]. The profound distal ischemia means that there is a maximal duration of use for REBOA that cannot be extended [22]. Periods of occlusion exceeding 40 min can result in irreversible organ injury and death. Additionally, supraphysiologic increases in blood pressure proximal to the occlusion balloon during REBOA can contribute to cardiac failure and exacerbation of traumatic brain injury [23].

Reinforcing the idea that the duration of the occlusion must be minimal, Saito N et al. reported that the time from inflation to deflation of the aortic balloon in 24-h survivors was shorter than in non-survivors. It was suspected that reperfusion injuries caused by systemic ischemia would lead to death. In experiments with swine, Morrison et al. reported that a longer aortic inflation time increased the release of interleukin-6, incidence of adult respiratory distress syndrome, and use of vasopressors [24].

In an attempt to minimize distal ischemia and extend the duration of use of REBOA, studies have led to the development of partial REBOA (pREBOA), whereby the balloon is deflated slightly, allowing a degree of flow beyond the balloon [21]. Several clinical and translational reports suggest that partial aortic flow restoration via partial aortic occlusion may serve to simultaneously mitigate the adverse effects of aortic occlusion on both proximal and distal vascular beds, whilst aiming to limit ongoing hemorrhage in the bleeding patient [25].

Although the REBOA technique continues to be studied, some studies demonstrate that a partial approach maintained normal physiology better than the complete one, minimized the systemic impact of distal organ ischemia, and reduced hemodynamic instability, allowing the potential for longer periods of intervention [23].

### Management during balloon occlusion

During balloon occlusion, specific complications can occur such as accessing the wrong vascular tree, misplacement of the wire or balloon within the arterial system, the creation of dissection flaps or other arterial injury, retroperitoneal hemorrhage, the development of lactic acidosis and organ dysfunction, and the development of clots which may lead to limb ischemia [1].

REBOA placement in some countries currently requires large arterial sheaths such as 7 to 14 Fr in the common femoral artery. It has been reported that these large sheaths may be associated with severe complications, including lower extremity ischemia and amputations. These complications may be related to the near occlusive diameter of these large sheaths, the length of time they remain in the artery, the location of insertion, and potential damage that can be caused during the insertion.

These problems related to the management of REBOA has led physicians to hypothesize that one of the causes associated with the rate of complications could be the diameter of the sheaths. A prospective observational study by Walter L. et al. proposed that the use of new low-profile devices could decrease vascular complications associated with REBOA [6].

A retrospective review of patients receiving REBOA through a 7 Fr sheath for refractory traumatic hemorrhagic shock performed from January 2014 to June 2015 at five tertiary-care hospitals in Japan reported that 7 Fr introducer device for REBOA may be a safe and effective alternative to large-bore sheaths and may remain in place during the post-procedure resuscitative phase without sequelae. The main benefits of a 7 Fr system include tolerance of a prolonged indwelling sheath time and the ability to remove the sheath successfully with only manual compression [5].

### Balloon deflation

REBOA balloon deflation and subsequent reperfusion is an integral stage in the procedure and can lead to potential cardiovascular complications. Previously, clinical guidelines have recommended the controlled deflation of the balloon to minimize sudden physiologic derangements. However, a study conducted on use of REBOA in 13 patients with pelvic fractures found six patients experienced hemodynamic shock upon balloon deflation. Of these six patients, three were resuscitated, one recovered after reinflation of the balloon, and the remaining two died from the shock [26]. This is thought to be due to the rapid release of ischemic metabolites such as nitric oxide and pro-inflammatory mediators after deflating the REBOA balloon, resulting in vasodilation and refractory hypotension, which ultimately leads to hemodynamic collapse [10]. Furthermore, adequate communication within the resuscitation team and with the anesthesia team is vital to ensure that preparations are in place for immediate reinflation of the balloon if needed. This approach attempts to prevent the rapid decrease in afterload and subsequent hypotension that can lead to hemodynamic instability [8]. However, an animal study conducted on eight swine models of hemorrhage found that graded balloon deflation still led to a rapid increase in aortic flow followed by a decrease in proximal mean arterial pressure. Furthermore, the time required for return of distal aortic flow was variable and inconsistent across the subjects [27].

### Sheath removal and post-operative management

After completion of the procedure and deflation of the balloon, both the REBOA balloon catheter (and wire if used) may be removed and various techniques may be employed to remove the device, ensuring there is no clot in the sheath or distal extremity of the sheath. The sheath can then be removed through a surgical longitudinal incision through the groin, exposing both distal and proximal areas to the sheath, before adequate closure of the artery [8]. In a 5-year retrospective study of 48 patients that underwent REBOA, the development of distal thrombus and arterial dissection was a common occurrence, due to the extended periods of occlusion after insertion of the sheath. Five patients required additional vascular procedures; two required thrombectomy with repair of the dissection flap and patch angioplasty; one required thrombectomy with patch angioplasty; one required thrombectomy, interposition graft, and prophylactic fasciotomy; and one required thrombectomy with repair of dissection flaps. None of these patients experienced any complications from the procedures [16]. Lower limb ischemia resulting in amputation has also been a reported complication following sheath removal. In a 6-year retrospective study conducted in Tokyo, Japan (*n* = 24), two patients experienced lower limb ischemia following sheath removal, both of which required amputation below the knee. This is resultant from the prolonged systemic ischemia [24]. The study also reported other major systemic complications, including nine patients who experienced acute kidney injury and nine patients with multi-organ failure; also complications of systemic ischemia [24]. The inflammatory sequelae of REBOA is not well understood, but these results mandate the need for aggressive and pre-emptive diagnosis and treatment of ischemic metabolites, clinical consequences of prolonged aortic occlusion, and unrecognized procedural vascular complications. Vigilant assessment of abdominal end organ and distal extremity perfusion is critical, and imaging access sites within 24–48 h of sheath removal is prudent.

### Areas for future research

The exact indications for REBOA remain uncertain [9]. Future studies should focus on which patient populations are suitable for receiving REBOA, as well as identifying the timeframe at which REBOA is most effective. Before the medical community seeks to widen the indications of REBOA, all of its complications should be understood first [1]. One of the current challenges to the widespread adoption of REBOA is a lack of data. More solid, prospective evidence of the complications at each stage of the REBOA is needed. A stronger evidence base for the complications at each stage of the procedure are needed to fully understand when and where REBOA is most effective as well as the conditions in which it should not be performed. With increasing use worldwide, more research and data will hopefully realize the great potential of REBOA not only for NCTH but also among a wider range of torso hemorrhage in trauma medicine.

## Discussion

### REBOA or no REBOA?

The use of endovascular aortic occlusion is an adjunct for resuscitation in patients with severe hemorrhage. In the setting of traumatic arrest from hemorrhage below the diaphragm, RT with cross-clamp may be used instead of REBOA for the purpose of aortic occlusion. In patients with physiologic decompensation, the advantage of REBOA is the ability to place the catheter at the intended level of occlusion, monitor the intra-aortic pressure with high-fidelity (if using the ER-REBOA catheter), and rapidly inflate the balloon prior to arrest is less invasive and committal than a RT. In the setting of severe pelvic hemorrhage, traditional control with pelvic packing and/or internal iliac ligation can be augmented by REBOA placed prior to these measures as a bridge to hemostasis. The benefits of using REBOA are largely based on the nature of it being a less invasive procedure and being able to intervene earlier in the downward spiral of exsanguinating hemorrhage: REBOA offers immediate, early temporization of hemorrhage prior to cardiovascular collapse. Consequences of early temporization may include decreased blood product transfusions with their inherent risks and sequela, less stress on cardiac function, decreased secondary brain injury for those with significant TBI, and the chance for survival beyond the ED. Significant research including the role of partial REBOA will help refine its use for a wide variety of clinical scenarios.

## Conclusion

REBOA is an emergent and increasingly accepted technique used as a less invasive alternative for controlling bleeding in patients with NTCH. However, for this procedure to be used in widespread practice, a better understanding of the potential complications that can arise in all stages must be well recognized. Complications can arise in arterial access, balloon positioning, inflation, during occlusion, deflation, and removal of the sheath. Comprehensive investigation and studies conducted into each of these stages of REBOA can allow identification of specific complications and adequate measures to be taken to avoid these complications and reduce potential morbidity and mortality associated with REBOA.

## Abbreviations

EVAR: Endovascular Management of Abdominal Aortic Aneurysms
NCTH: Non-compressible torso hemorrhage
pREBOA: Partial REBOA
rAAA: Ruptured abdominal aortic aneurysm
REBOA: Resuscitative Endovascular Balloon Occlusion of the Aorta
RT: Resuscitative thoracotomies
